# Supplementary material for: Genomic and Epigenomic Changes in the Progeny of Cold-Stressed Arabidopsis thaliana Plants
Source: Int J Mol Sci. 2024 Feb 28;25(5):2795. doi: 10.3390/ijms25052795 (PMC10932141; doi:10.3390/ijms25052795)
Supplement: Supplementary file 1 [file ijms-25-02795-s001.zip › ijms-2808395-supplementary.pdf]

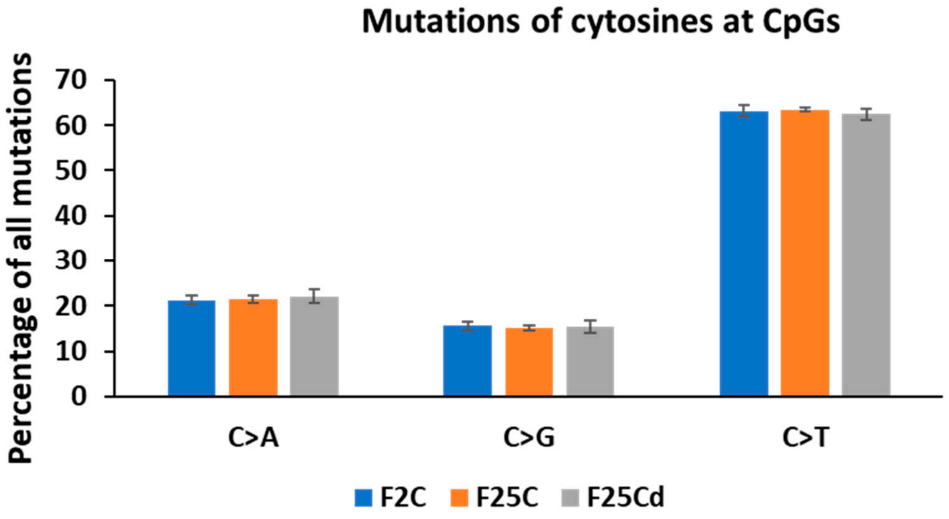

Figure S1. Type of nucleotide substitutions in F2C, F25C and F25Cd groups at cytosines at the CpG (cytosine followed by guanine) sites. Y axis shows the percentage of mutations of certain type, while X axis shows the group. Data are averaged (with SE) from five individual plants. A – adenine, C – cytosine, G – guanine, T – thymine.

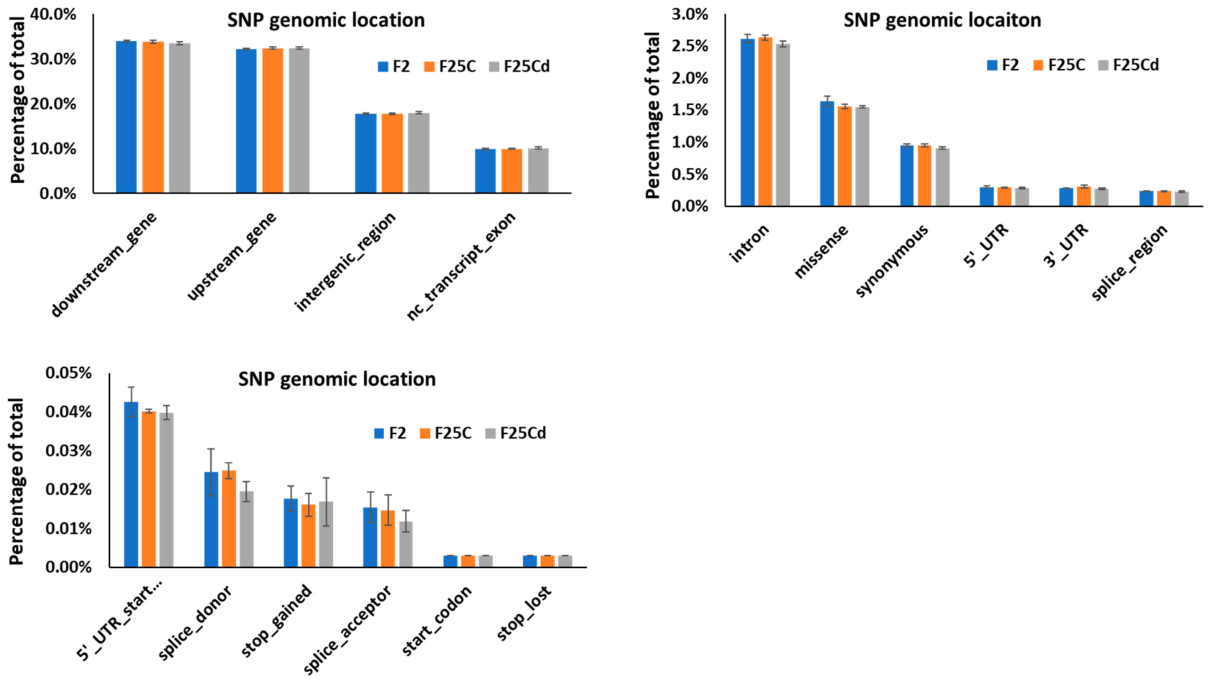

Figure S2. The distribution of variants across the genome by their potential effects based on genomic positions. The percentage of variant effects (from total) is shown as an average calculated from five samples (with SD), and genomic positions effects from SnpEff are listed on the X-axis. Data are shown in three different graphs for convenience.

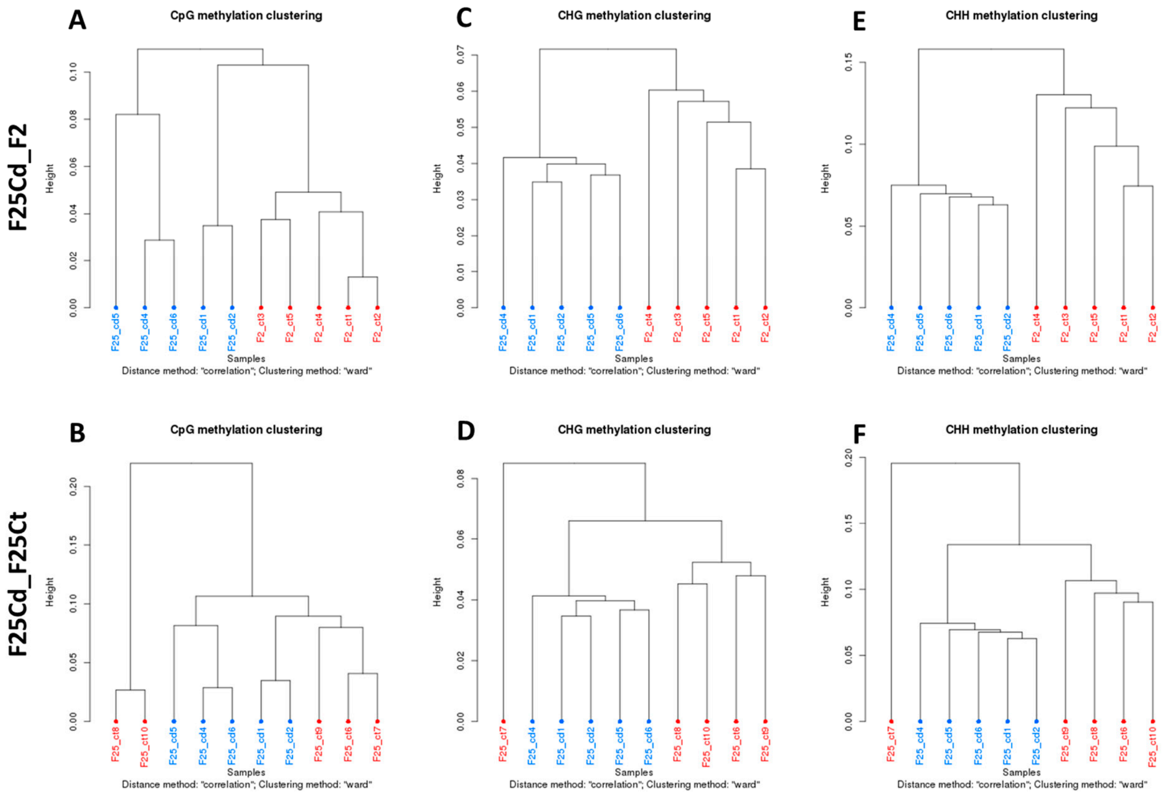

Figure S3. CpG (A, B), CHG (C, D) and CHH (E, F) methylation clustering of differentially methylated regions (DMRs, 100 bp window) in the F25Cd vs F2 (A, C, E) and F25Cd vs F25C (B, D, F) comparison groups. CpG (cytosine followed by guanine), CHG (cytosine followed by any nucleotide, followed by guanine), and CHH (cytosines followed by any nucleotides). Hierarchical clustering of all fifteen methylomes was done by using Pearson's correlation distance.

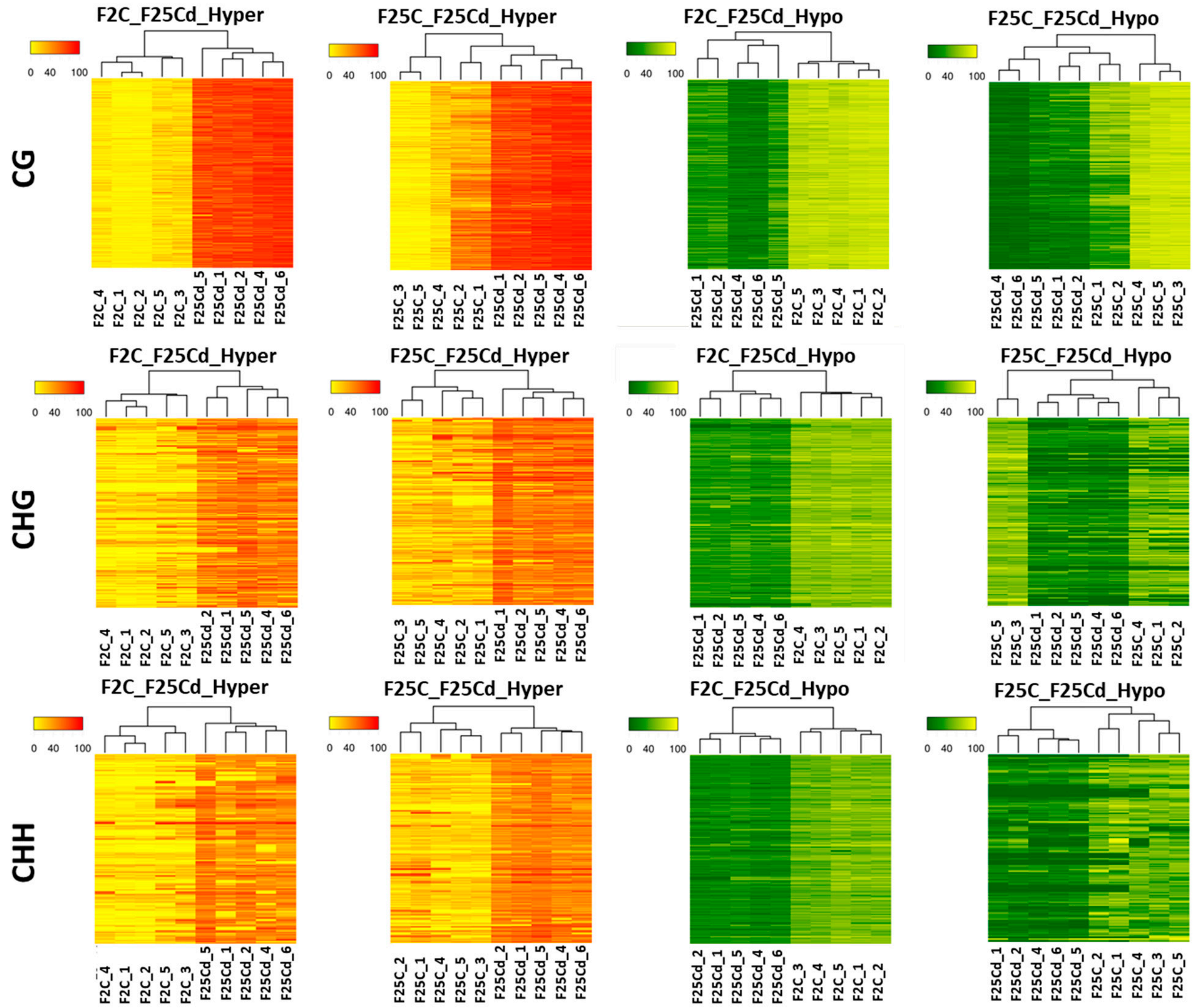

Figure S4. A hierarchical clustering heatmap analysis of differentially methylated cytosines (DMCs). Heat maps of DMCs for hypermethylated cytosines (the upper panel) and hypomethylated cytosines (the lower panel) in CpG (cytosine followed by guanine), CHG (cytosine followed by any nucleotide, followed by guanine), and CHH (cytosines followed by any nucleotides) contexts in F25Cd vs F2, F25C vs F2C and F25Cd vs F25C comparison groups, shown for positions with > 50% difference (q-value <0.01).

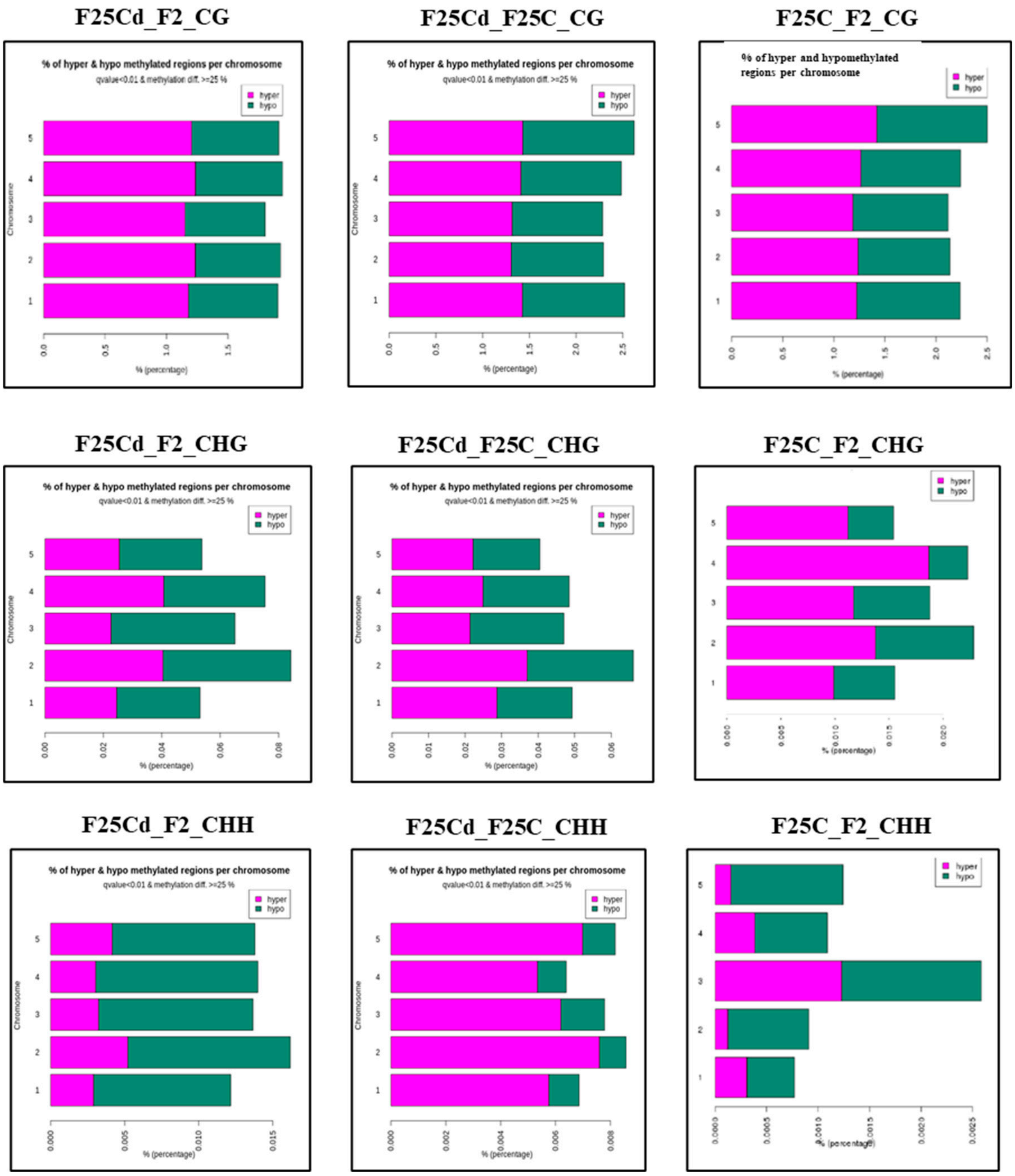

Figure S5. The distribution of differentially methylated regions (DMRs) across the chromosomes. Distributions of differentially methylated regions are shown in the CpG (upper panel), CHG (middle panel) and CHH (lower panel) contexts in a 100-bp window. CpG (cytosine followed by guanine), CHG (cytosine followed by any nucleotide, followed by guanine), and CHH (cytosines followed by any nucleotides). The horizontal bar plots show the number of methylated events per chromosome as a percent of sites with minimum coverage and differential. The pink section indicates the percentage of hypermethylation, and the green one indicates hypomethylation, q-value <0.01, and methylation difference > 25%.

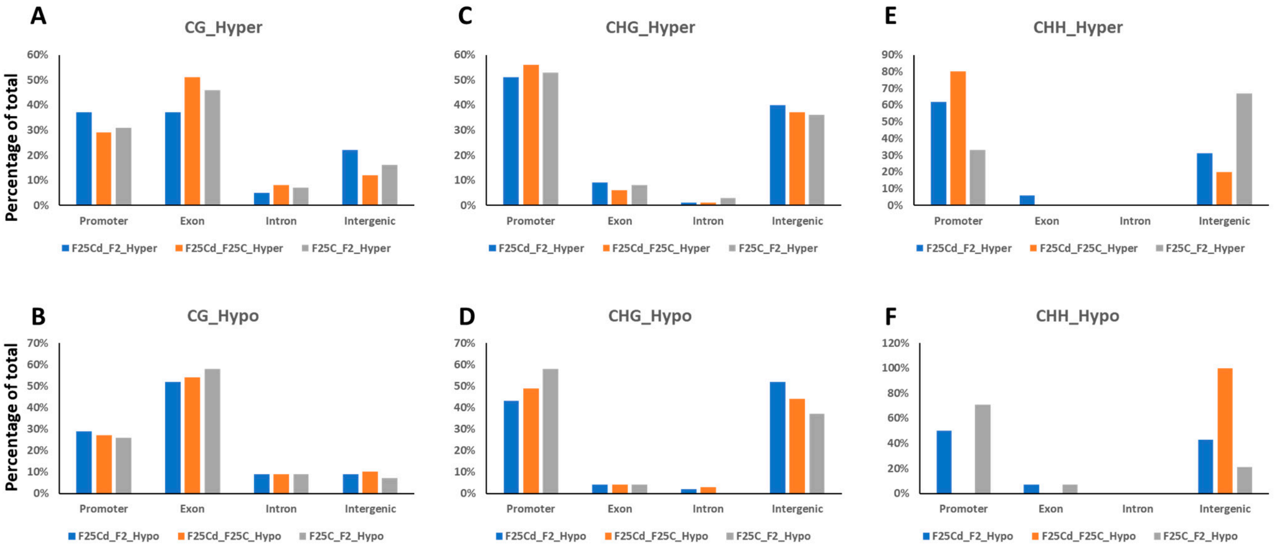

Figure S6. The percentages of differentially hypermethylated and hypomethylated differentially methylated regions (DMRs, 100 bp window) in CG (A, B), CHG (C,D) and CHH (E,F) contexts in different genomic regions in F2C vs. F25Cd, F25C vs. F25Cd, and F2C vs. F25C comparison groups. CpG (cytosine followed by guanine), CHG (cytosine followed by any nucleotide, followed by guanine), and CHH (cytosines followed by any nucleotides). The percentages plotted are the average percentages of DMRs overlapping various genomics regions, including promoters, exons, introns, and intergenic regions where DMRs were considered as regions with > 25% difference in methylation with the coverage of at least 10 sequence reads per DMR.

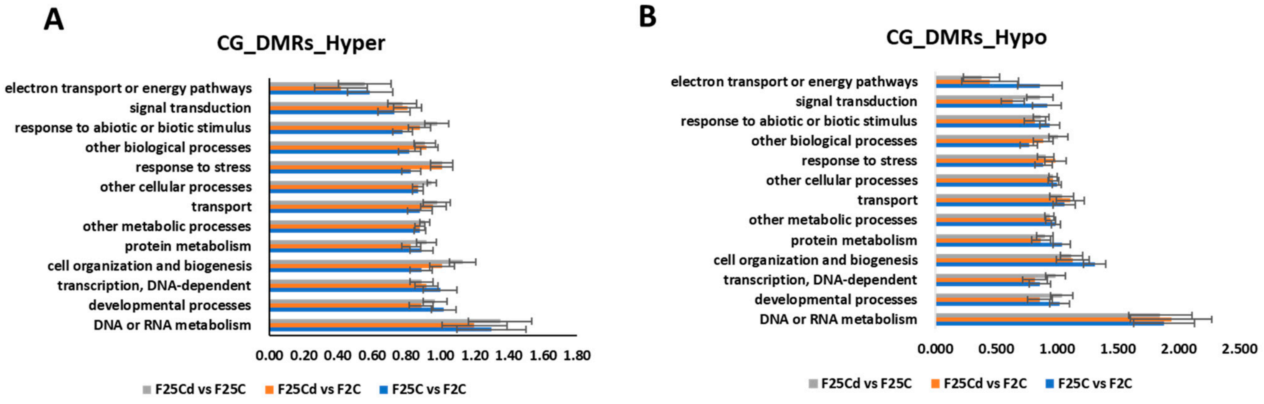

Figure S7. The enrichment analysis of hypermethylated (A) and hypomethylated (B) differentially methylated regions (DMRs) in the CG (cytosine followed by guanine) contexts and their classification based on the biological processes. The Y-axis shows significantly enriched biological processes. The X-axis is the normalized frequency with binomial coefficients as calculated by SuperViewer. Genes are classed with p-values < 0.05, ±bootstrap StdDev.
